# Quality of life trajectories for different dialysis modalities—a nationwide study

**DOI:** 10.1093/ckj/sfae420

**Published:** 2024-12-20

**Authors:** Helena Rydell, Aurora Caldinelli, Jenny Wrackefeldt, Aline Kåveryd-Hult, Bengt Lindholm, Abdul Rashid Qureshi, Nicholas C Chesnaye, Marie Evans

**Affiliations:** Renal Medicine, Department of Clinical Science, Intervention and Technology (CLINTEC), Karolinska Institutet, Stockholm, Sweden; Renal unit, Karolinska University Hospital, Stockholm, Sweden; Swedish Renal Registry, Jönköping, Sweden; Department of Medical Epidemiology and Biostatistics (MEB), Karolinska Institutet, Solna, Sweden; Renal Medicine, Department of Clinical Science, Intervention and Technology (CLINTEC), Karolinska Institutet, Stockholm, Sweden; Renal unit, Karolinska University Hospital, Stockholm, Sweden; Swedish Renal Registry, Jönköping, Sweden; Transplant Center, Sahlgrenska University Hospital, Gothenburg, Sweden; Renal Medicine, Department of Clinical Science, Intervention and Technology (CLINTEC), Karolinska Institutet, Stockholm, Sweden; Renal Medicine, Department of Clinical Science, Intervention and Technology (CLINTEC), Karolinska Institutet, Stockholm, Sweden; ERA Registry, Amsterdam UMC location University of Amsterdam, Medical Informatics, Meibergdreef 9, Amsterdam, The Netherlands; Amsterdam Public Health Research Institute, Quality of Care, Amsterdam, The Netherlands; Renal Medicine, Department of Clinical Science, Intervention and Technology (CLINTEC), Karolinska Institutet, Stockholm, Sweden; Renal unit, Karolinska University Hospital, Stockholm, Sweden; Swedish Renal Registry, Jönköping, Sweden

**Keywords:** home hemodialysis, in-center hemodialysis, peritoneal dialysis, quality of life, RAND-36

## Abstract

**Background:**

Few contemporary studies have investigated the changes in quality of life across dialysis modalities. Our aim was to compare longitudinal changes in health-related physical and mental quality of life between patients on institutional hemodialysis (IHD), peritoneal dialysis (PD) and home hemodialysis (HHD).

**Methods:**

Patients on dialysis with registered Research and Development 36 (RAND-36) questionnaires between 2017–2021 in the Swedish Renal Registry (SRR) were eligible for inclusion. Information on dialysis, patient characteristics and medication were collected from SRR and other registries. Patients were followed up to 39 months. Changes in physical (PCS) and mental (MCS) component summary scores were analyzed in adjusted linear mixed models and joint models.

**Results:**

We included 930 patients (IHD 714, PD 128, HHD 88) with a median follow-up of 1.8 years (interquartile range 1.0–2.1). At baseline, the mean unadjusted PCS was lower in IHD (30.7; 95% CI 29.9, 31.4) compared with HHD (35.3; 95% CI 33.0, 37.5) and PD (33.2; 95% CI 31.3, 35.1). PCS declined over time in all modalities, but faster for PD compared with IHD (–1.2; 95% CI –2.1, –0.3 per year) and HHD (–1.5, 95% CI –2.9, –0.04). MCS was similar at baseline. HHD had improving MCS trajectory compared to IHD (–1.5, 95% CI –2.8, 0.2) and PD (–2.3, 95% CI –3.9, 0.7), largely resulting from improvement in role limitations caused by mental health (6.2, 95% CI 0.9, 11.5).

**Conclusion:**

Insights about the variation in quality-of-life trajectories for different dialysis modalities are important for patients to make informed choices.

## Graphical Abstract

**Graphical Abstract:**
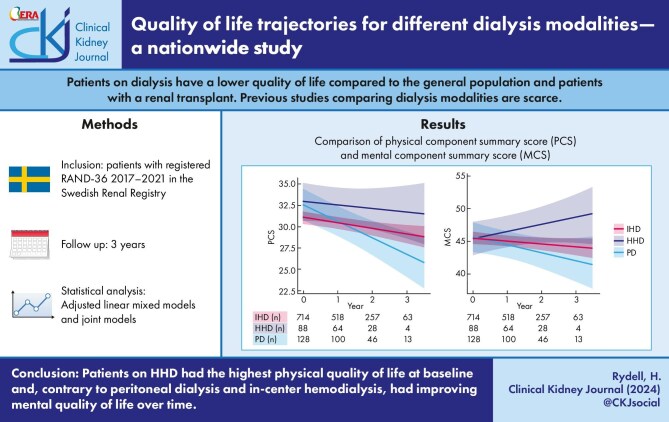

KEY LEARNING POINTS **What was known:**

- Most studies comparing quality of life in patients on different dialysis modalities have been cross-sectional, small, old, short-term and none of them incorporated analysis of differences in informative drop-out during the follow-up. Moreover, there's a paucity of evidence regarding changes in quality of life in patients on home hemodialysis.

**This study adds:**

- Patients on home hemodialysis had higher physical quality of life at baseline and, contrary to peritoneal dialysis and in-center hemodialysis, improving mental quality of life trajectory over time.
- Physical quality of life was higher for patients on peritoneal dialysis than in-center hemodialysis at baseline but decreased faster over time compared with home and in-center hemodialysis.

**Potential impact:**

- This information is pertinent to patients to be able to make informed choices between dialysis modalities. Offering home hemodialysis to all patients who are able and willing to perform self-care dialysis could potentially improve the quality of life for patients on dialysis.

## INTRODUCTION

In addition to high mortality and morbidity [1, 2], patients on dialysis have a lower quality of life compared with the general population [3, 4] and patients with a kidney transplant [3]. Quality of life is an important determinant for patients when

planning for future dialysis care [5]. The choice of treatment modality not only influences the ability to continue working, keep up personal or social relationships, travel, and maintain freedom, it also affects other domains of the patients’ well-being such as sleep and recovery time [6].

Although there is some information about quality of life for dialysis patients, the evidence that could inform patients and health professionals about changes in quality of life over time on dialysis is scarce [7, 8]. Most studies comparing quality of life in dialysis patients of different modalities have been cross-sectional [8–18], small [9–11, 14, 15, 18–25], old [17, 19, 26, 27], short-term [13, 22, 26, 28] and none of the previous studies incorporated differences in informative drop-out during the follow-up in their statistical analysis [7]. Taking informative censoring into account is important because the characteristics of patients initiating peritoneal dialysis (PD) or in-center hemodialysis (IHD) are different, and hence the reasons for drop-out. Additionally, there is a paucity of evidence regarding changes in quality of life in patients on home hemodialysis (HHD). In this study, we therefore aim to compare the longitudinal trajectories in mental and physical components of quality of life between patients on IHD, PD, and HHD, while taking informative drop-out into account.

## MATERIALS AND METHODS

### Study population

We used data from the Swedish Renal Registry (SRR). All patients on dialysis were eligible for inclusion if they had a registered Research and Development-36 (RAND-36) questionnaire in SRR 2017–2021 and were included in at least one yearly cross-sectional dialysis survey, which takes part every year between 15 September and 15 October. All patients on chronic dialysis in Sweden participate in this survey, which includes registration of clinical characteristics, dialysis data and laboratory measurements. Clinics are also encouraged to perform a RAND-36 evaluation at the time of the survey. Patients with at least two registered RAND-36 questionnaires were followed from the first questionnaire (index date) until death, kidney transplantation, 31 December 2021, or a maximum of 39 months. Patients who switched modality between the index date and the first cross-sectional survey were excluded. Data from the SRR were linked to other national registries (the National Patient Register, the National Prescribed Drug Register, and the National Cause of Death Register) using unique personal identification numbers. The study was approved by the ethics committee (EPN Stockholm 2018-1591-31 and 2020-04 778).

### Definition of dialysis modality

The patient population was divided into three categories based upon the dialysis modality recorded at the first cross-sectional dialysis survey: IHD, PD and HHD. In Sweden, a proportion of the patients on hemodialysis perform the dialysis completely autonomously at a satellite or an in-center dialysis unit—this is referred to as self-hemodialysis or in-center HHD. The reason for patients not to bring home the hemodialysis treatment varies, but often depends on social circumstances such as the ability to own a house or apartment suitable for HHD, or a wish to separate the disease from family life. Regardless, these patients often perform their dialysis more frequently, and it is also possible to have more individualized schedules. For this investigation, patients on in-center HHD were categorized into the HHD group, whereas patients who require *any* assistance (self-hemodialysis with limited care) were categorized into the IHD group.

### Health-related quality of life

RAND-36 comprises 36 items that could be combined into eight dimensions (physical functioning, role limitations caused by physical problems role, bodily pain, general health, vitality/energy/fatigue, social functioning, mental health/emotional well-being, and role limitations caused by mental health/emotional problems) and two summary scales providing the physical component summary score (PCS) and the mental component summary score (MCS) [29, 30]. RAND-36 was first introduced in the SRR in 2017 [29]. The recommendation is that all patients on dialysis should fill out the questionnaire once yearly, either on paper or digitally at the same time as the annual cross-sectional dialysis survey [31]. Paper forms were entered manually into SRR by the local staff; no missing values were allowed, and only complete forms were uploaded.

The primary outcome in this study was the change in quality of life over time measured by the two summary scales of RAND-36. PCS and MCS were calculated using norm-based scoring, which uses linear transformation to achieve standardized scores with a mean (SD) of 50 (10) for each dimension by using the US population as a reference group [32]. Secondary outcomes were the difference in PCS and MCS at baseline and the change over time in the eight dimensions included in RAND-36.

### Covariates

Primary and secondary diagnoses from hospital admissions and specialist outpatient visits coded according to the International Classification of Diseases (ICD)-10 were used to obtain information about comorbid diseases diagnosed prior to the index date. Charlson comorbidity index and the number of days admitted to hospital during one year before index date were recorded from the National Patient Register using all ICD-10 codes and dates of hospital admissions [33, 34]. Information on drug dispensations from pharmacies according to the Anatomic Therapeutic Chemical classification system was collected from the National Prescribed Drug Register (dispensations within six months before the index date), whereas mortality was retrieved from the National Cause of Death Register. Age, sex, categories of primary kidney disease, dates of start and changes of kidney replacement therapies were all attained from the SRR, whereas laboratory values were recorded from the dialysis survey closest in time to the RAND-36 questionnaire.

### Statistical analysis

Categorical variables were reported as numbers and percentages, while continuous variables were reported as median and interquartile range (IQR), stratified by dialysis modality. Baseline values and change over time in PCS and MCS, overall and by dialysis modality, were first analyzed in a linear mixed regression model. Based upon a priori knowledge of potential confounders, we then adjusted the models for sex, age, Charlson comorbidity index score (categorical) score, diabetes mellitus, dialysis vintage, the number of days admitted to hospital during the year prior to index date, and laboratory values (plasma albumin, hemoglobin, phosphate). For the main analysis, our exposure (dialysis modality) was analyzed using an intention-to-treat approach. Patients were censored at the date of kidney transplantation. We tested the linear assumption using generalized additive models with smoothing splines and found no evidence of a better fit. Second, a joint model was used. Joint models are used to model time to event data and repeated measurement trajectories jointly. These types of models combine time-to-event competing risk analyses with linear regression models; in the present study these were used to account for informative censoring due to differences in the occurrence of death and kidney transplantation between groups [35]. In the time-to-event competing risk model we treated death as the informative censoring event and considered renal transplantation as a competing event. For most variables, there were no missing data (Supplementary Table S1). Adjusted analyses were conducted on complete cases.

We performed several sensitivity analyses. First, we included all patients with at least one RAND-36 measurement repeating both the linear mixed models and the joint models with follow up over 39 months. Second, we performed sensitivity analyses excluding patients who changed dialysis modality during the follow-up period (Supplementary Tables S9 and S10), and lastly we performed an analysis in patients with dialysis vintage below two years at baseline (Supplementary Table S11).

## RESULTS

We included 930 patients with at least two RAND-36 measurements (Supplementary Fig. S1). The patient characteristics are presented in Table 1. IHD was the most prevalent dialysis modality (*n* = 714, 77%) followed by PD (*n* = 128, 14%) and HHD (*n* = 88, 9%). Patients on HHD were younger, had diabetic nephropathy and nephroangiosclerosis as primary kidney disease less often, had lower Charlson comorbidity index and higher serum albumin indicating a better overall health status compared with IHD and PD. Patients on IHD were using more antidepressants compared with patients on HHD and PD. The dialysis vintage at start of follow-up was shorter for patients on PD compared with patients on HHD and IHD. Comparing the patient characteristics with the overall SRR population demonstrated small differences (Supplementary Table S12).

**Table 1:** Patient characteristics at the time of the first RAND-36 questionnaire, stratified by dialysis modality.

**Characteristics**	Total (*n* = 930)	IHD (*n* = 714)	HHD (*n* = 88)	PD (*n* = 128)
Age, years (IQR)	71.0 (59.0–78.0)	72.0 (61.0–79.0)	55.5 (46.8–63.0)	71.0 (58.0–77.0)
Female	310 (33%)	239 (33%)	32 (36%)	39 (30%)
Dialysis vintage, years	1.3 (0.6–3.4)	1.4 (0.7–3.6)	1.9 (0.8–5.5)	0.7 (0.3–1.6)
Days admitted to hospital year before index	10 (0–17.0)	6.0 (0.0–19.0)	2.0 (0.0–10.3)	3.0 (0.0–10.3)
Days admitted to hospital year before index	10 (0–17.0)	6.0 (0.0–19.0)	2.0 (0.0–10.3)	3.0 (0.0–10.3)
**Primary kidney diseases**				
Polycystic kidney disease/hereditary	70 (7.5%)	48 (6.7%)	8 (9.1%)	14 (11%)
Diabetic nephropathy	214 (23%)	174 (24%)	16 (18%)	24 (19%)
Glomerulonephritis	164 (18%)	118 (17%)	22 (25%)	24 (19%)
Nephroangiosclerosis	186 (19.8%)	145 (20.7%)	10 (11.1%)	31 (23.8%)
Other	31.7 (%)	31.6 %	36.8 %	27.2 %
**Laboratory values**				
Hemoglobin (g/l)	114.0 (106.0–122.0)	114.0 (106.0–121.3)	113.5 (106.8–122.0)	113.5 (107.0–125.0)
Phosphate (mmol/l)	1.6 (1.3–1.9)	1.6 (1.3–1.9)	1.6 (1.4–2.0)	1.5 (1.3–1.8)
Albumin (g/l)	35.0 (32.0–37.0)	35.0 (32.0–37.0)	36.0 (33.0–39.0)	31.0 (28.0–34.3)
C-reactive protein (mmol/l)	5.0 (2.8–12.0)	5.0 (2.8–12.0)	5.0 (2.9–9.0)	5.0 (3.0–8.0)
**Comorbidity**				
*Median (IQR)*	3 (2–4)	3 (2–4)	2 (1–4)	2 (1–4)
Coronary artery disease	281 (30%)	230 (32%)	17 (19%)	34 (27%)
Congestive heart failure	309 (33%)	258 (36%)	23 (26%)	28 (22%)
Peripheral vascular disease	131 (14%)	106 (15%)	13 (15%)	12 (9.4%)
Cerebrovascular disease	176 (19%)	145 (20%)	12 (14%)	19 (15%)
Dementia	4 (0.4%)	4 (0.6%)	0 (0%)	0 (0%)
Use of antidepressant	160 (17%)	132 (18%)	13 (15%)	15 (12%)

Values are presented as number (%) for categorical variables and median (IQR) for continuous variables.

IHD, in-center hemodialysis; HHD, home-hemodialysis, PD, peritoneal dialysis.

Patients were followed for a median of 1.8 years (IQR 1.0–2.1) with a mean number of 2.6 (95% CI 2.5, 2.6) RAND-36 measurements. In total 201 (22%) patients died and 75 (8%) received a kidney transplant during the follow-up period. Mortality was highest among patients undergoing IHD (23%), followed by PD (19%) and HHD (17%), whereas the proportion of patients who received a transplantation was highest for the HHD group followed by PD and IHD (Supplementary Table S2).

### Physical component summary score and mental component summary score at baseline

Patients on IHD had the lowest PCS at baseline (30.7; 95% CI 29.9, 31.4), whereas the absolute values of PCS at baseline for HHD and PD were 35.2 (95% CI 33.0, 37.5) and 33.2 (95% CI 31.3, 35.1), respectively (Table 2). However, the differences in PCS were attenuated in the adjusted model and failed to reach statistical significance.

**Table 2:** Physical component summary score and mental component summary score at baseline in patients on IHD and HHD or PD.

	Linear mixed model					
	Unadjusted		Adjusted*		Joint model Adjusted*	
	Mean score (95% CI)	*P*-value*	Mean score (95% CI)	*P*-value*	Mean score (95% CI)	*P*-value*
Physical component summary score						
IHD (ref)	30.7 (29.9, 31.4)	<.0001	37.0 (29.2, 44.8)	<.0001	37.5 (30.1, 44.8)	<.0001
HHD	35.2 (33.0, 37.5)	.0002	38.8 (30.9, 46.7)	.13	39.0 (31.5, 46.4)	.18
PD	33.2 (31.3, 35.1)	.01	38.4 (30.8, 46.1)	.16	38.8 (31.4, 46.1)	.18
Mental component summary score						
IHD (ref)	45.6 (44.7, 46.5)	<.0001	34.1 (24.9, 43.1)	<.0001	33.9 (25.2, 42.7)	<.0001
HHD	43.8 (41.2, 46.4)	.21	34.09 (24.9, 43.3)	.99	33.9 (24.9, 42.9)	.98
PD	45.6 (43.5, 47.8)	.97	34.2 (25.4, 43.2)	.87	34.2 (25.8, 42.6)	.81

HHD, home-hemodialysis; IHD, in-center hemodialysis; PD, peritoneal dialysis.

Adjusted for sex, age, Charlson Comorbitiy Index score, diabetes, dialysis vintage, the number of days admitted to hospital the last year before baseline, and laboratory values (albumin, hemoglobin, phosphate).

*P-value compared to IHD.

The baseline MCS for IHD patients was 45.6 (95% CI 44.7, 46.5) whereas the corresponding absolute values for HHD and PD were 43.8 (95% CI 41.2, 46.4) and 45.6 (95% CI 43.5, 47.8), respectively. There was no statistically significant difference in the MCS for HHD or PD at baseline as compared to MCS for IHD (Table 2) in the unadjusted or adjusted models.

### Change over time in physical component summary

**Figure 1:**
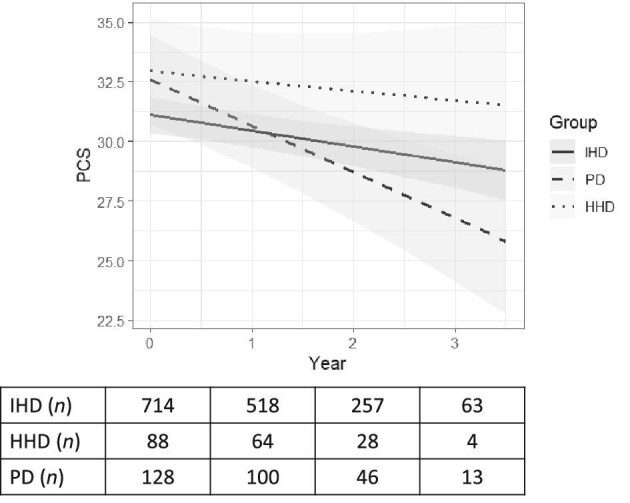
Comparison of physical component summary score (PCS; estimated marginal means) of quality of life between patients on IHD, HHD and PD. *Linear mixed adjusted model (sex, age, Charlson Comorbidity Index score, diabetes, dialysis vintage, the number of days admitted to hospital the last year before baseline, and laboratory values; albumin, hemoglobin, phosphate).

Overall, the PCS trajectory declined over the follow-up period. The absolute unadjusted yearly change in PCS was –0.6 (95% CI –0.9, –0.2), –0.4 (95% CI –1.5, 0.6) and –1.9 (95% CI –2.8, –1.0) for patients on IHD, HHD and PD, respectively. In the adjusted linear mixed model, PCS values in patients on IHD declined by –0.6 (95% CI –1.0, –0.2) per year and –0.9 (–1.3, –0.5) per year in the joint model (Table 3; Fig. 1). The longitudinal trend for HHD patients indicated a slower decline in PCS compared to IHD patients (0.4 points per year; 95% CI –0.7, 1.5), although this difference was not statistically different (Table 3). PCS declined faster in PD patients compared with patients on IHD (Table 3) and HHD (Supplementary Table S3), both in the linear mixed models and the joint models (–1.2 points compared to IHD; 95% CI –2.1, –0.3; and –1.5 points compared to HHD; 95% CI –2.9, –0.04).

**Table 3:** Differences in the changes over time in physical component summary score and mental component summary score in patients on IHD and HHD or PD.

	Linear mixed model		
	Unadjusted	Adjusted*	Joint model Adjusted*
	Estimate	Estimate	Estimate
	(95% CI)	(95% CI)	(95% CI)
Physical component summary score			
IHD (ref)	–0.6 (–0.9, –0.2)	–0.6 (–1.0, –0.2)	–0.9 (–1.3, –0.5)
HHD^a^	0.2 (–0.98, 1.3)	0.2 (–0.9, 1.4)	0.4 (–0.7, 1.5)
PD^a^	–1.3 (–2.3, –0.3)*	–1.3 (–2.3, –0.3)*	–1.2 (–2.1, –0.3)*
Mental component summary score			
IHD (ref)	–0.4 (–0.9, 0.05)	–0.4 (–0.9, 0.04)	–0.5 (–0.9, –0.08)
HHD^b^	1.5 (0.1, 3.0)*	1.5 (0.1, 2.9)*	1.5 (0.1, 2.8)*
PD^b^	–0.8 (–2.1, 0.4)	–0.8 (–2.0, 0.5)	–0.8 (–1.9, 0.3)

HHD, home-hemodialysis; IHD, in-center hemodialysis; PD, peritoneal dialysis.

Adjusted for sex, age, Charlson Comorbidity Index score, diabetes, dialysis vintage, the number of days admitted to hospital the last year before baseline, and laboratory values (albumin, hemoglobin, phosphate).

*P-value < 0.05 compared to IHD.

^a^Mean difference in score compared to the reference (IHD).

^b^Mean difference in change/year compared to reference (IHD). A positive value indicates a slower change; a negative value indicates a faster change/year.

### Change over time in mental component summary

**Figure 2:**
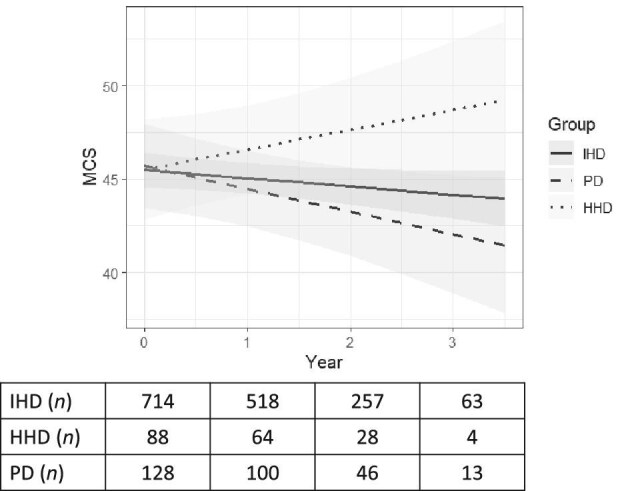
Comparison of mental component summary score (MCS; estimated marginal means) of quality of life between patients on IHD, HHD and PD. *Linear mixed adjusted model (sex, age, Charlson Comorbidity Index score, diabetes, dialysis vintage, the number of days admitted to hospital the last year before baseline, and laboratory values; albumin, hemoglobin, phosphate).

The MCS trajectory declined over the follow-up for patients on IHD and PD but increased for patients on HHD. The absolute unadjusted yearly change in MCS was –0.4 (95% CI –0.9, 0.05), 1.1 (95% CI –0.2, 2.5) and –1.2 (95% CI –2.4, –0.1) for patients on IHD, HHD and PD, respectively. MCS decreased over time in patients on IHD in both the adjusted mixed model and was significantly different from no change in the joint model (–0.5; 95% CI –0.9, –0.08 Table 3, Fig. 2). Patients on HHD had improving MCS over time that reached statistical significance compared to IHD (Table 3) and PD (Supplementary Table S4) in both the unadjusted and adjusted linear mixed model and joint model (1.5 compared to IHD; 95% CI 0.1, 2.8). There was no statistical difference in change of MCS for patients on PD (–0.8; 95% CI –1.9, 0.3) compared to IHD.

### Change over time in the eight dimensions included in RAND-36

**Figure 3:**
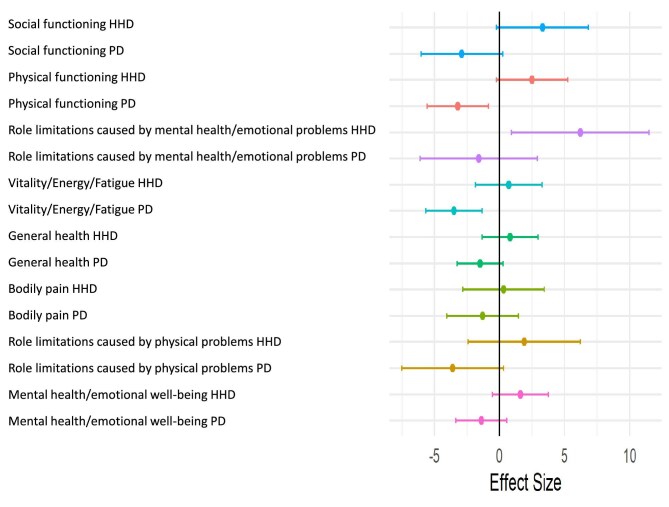
Difference between IHD versus PD or HHD over follow-up in the eight dimensions included in the RAND-36. *Linear mixed adjusted model (sex, age, Charlson Comorbidity Index score, diabetes, dialysis vintage, the number of days admitted to hospital the last year before baseline, and laboratory values; albumin, hemoglobin, phosphate).

The mean difference over time between IHD, HHD and PD for the eight dimensions of RAND-36 is shown in Fig. 3 and Supplementary Table S5. There was a trend towards higher values for HHD compared to IHD in several of the dimensions, but a statistically significant difference compared to IHD was only obtained for role limitations caused by mental health/emotional problems [6.2 points (95% CI 0.9, 11.5)]. There was also a trend towards lower values for PD compared to IHD; a statistically significant difference was observed in change of physical functioning in PD [–3.2 points (95% CI –5.6, –0.7)] and vitality [–3.5 points (95% CI –5.6, –1.3)] compared to patients on IHD.

### Sensitivity and supporting analyses

The sensitivity analyses in the cohort of 2137 patients with at least one RAND-36 measurement (Supplementary Tables S6-8) were congruent with the main results, but some differences between groups did not reach statistical significance*.* In the sensitivity analysis excluding patients who changed dialysis modality during follow-up, we observed that the magnitude of the difference in PCS trajectory between patients on PD and IHD decreased and were no longer statistically significant, whereas the difference between MCS trajectory for HHD and IHD patients increased. The sensitivity analysis excluding patients with a longer dialysis vintage, was consistent with the main results although confidence intervals became wider, and some associations lost statistical significance.

## DISCUSSION

In this contemporary cohort study of patients on maintenance dialysis in Sweden, we demonstrate that there are differences in patients’ health-related quality of life between the dialysis modalities both at baseline and over time. Although we cannot rule out that our results were influenced to some extent by non-measured differences such as health literacy and socioeconomic status, which might be related to the ability to perform self-administered dialysis regimes, we found that patients on HHD had higher PCS at baseline compared with IHD and that HHD patients were the only group with an increasing MCS trend over time.

The physical function declined over time for all dialysis modalities, but the decline was faster for patients on PD compared with IHD and HHD, and also after adjustments for underlying patient characteristics and informative drop-out.

The differences between HHD and the other modalities are larger compared to the differences previously shown in health-related quality of life between patients with other severe chronic diseases and the general population [36] and from that perspective are clinically highly relevant. However, there is a broad range in the reported minimal clinically important changes in health-related quality of life in the previous literature with focus on patients with chronic kidney disease (CKD), between 2 and 9 measured by RAND-36 or SF-36 [37]. It differs between contexts and groups of patients. The higher values in patients with CKD are reported for patients with CKD 5 and conservative care, thus patients with higher age and comorbidity burden compared to the population in the present study [37].

Attempts have been made to investigate differences in quality of life between HD and PD [8, 38]. Most cross-sectional comparisons, based on a variety of questionnaires, indicated somewhat higher scores for PD patients as compared to HD, that is mainly IHD [9, 12, 15, 16, 38]. At baseline, our results also demonstrated higher mean PCS scores for PD than for IHD patients, although not statistically different after adjustments. There are also a small number of previous prospective cohort studies with sample sizes ranging from 56 to 989 patients [7, 24]. Although most studies were short-term [13, 19, 26, 39], some had a follow-up of up to two years [20, 23, 25, 39, 40]. Contrary to our results, four of the previous prospective cohort studies saw no overall differences in the quality of life for PD versus IHD [23, 26, 27, 41], whereas three others favored PD over IHD [13, 20, 39]. There could be several reasons for these divergent results, but it is likely that heterogeneity in study designs, comparators, and differences in case-mix are possible explanations. It is well known that patient characteristics could be quite different for IHD and PD patients; PD patients are often younger and healthier, and without careful adjustment there may be residual confounding influencing the results. In Sweden, PD is initiated in a moderately large proportion of incident patients (about 30%), and the mean age is increasing and approaching that of IHD patients (Table 1) [4].

Contrary to some of the earlier studies, we did not observe any difference in the quality of life between patients on PD and IHD at baseline, and the decline in physical function and vitality over time was faster for patients with PD. It is possible that in our study with longer follow-up, these changes may to some extent reflect a diminishing residual kidney function and with time inadequate increase of dialysis doses. We also need to consider that other studies have shown a higher treatment satisfaction with PD compared to IHD [40, 41], something that we did not measure in our analysis. Moreover, in the sensitivity analysis including patients who remained in the same dialysis modality throughout follow-up, we observed a less-rapid decline in the PCS trajectory for PD patients in relation to IHD patients with no statistical difference. This might indicate that it is the patients who no longer benefit from PD who change to hemodialysis, and for those who remain in PD, there is no or little difference in PCS trajectory as compared to IHD.

The comparison in our study of longitudinal changes in the quality of life in HHD patients with corresponding changes in patients on IHD and PD is novel. However, a few previous studies evaluated quality of life in HHD patients with a focus on dialysis frequency. One older trial compared nocturnal HHD to conventional hemodialysis, 4 hours 3 times weekly performed as HHD or IHD [7, 27, 42]. In this small study including 52 patients, frequent nocturnal hemodialysis was associated with improvements in some specific kidney-specific domains of quality of life, as well as burden of kidney disease. Only a non-significant trend towards higher overall quality of life for patients with nocturnal HHD was observed. More recently, the Frequent Hemodialysis Network group compared daily in-center hemodialysis with conventional hemodialysis, 4 hours 3 times weekly, and found shorter recovery times after dialysis and a higher quality of life for patients on frequent hemodialysis [43]. In our study, HHD patients as compared with PD and IHD patients had superior quality of life with a higher PCS at baseline and an increasing MCS over time. This could be related both to the autonomy that is inherent in self-administered dialysis and to regimes with more frequent dialysis and higher dialysis doses. Among patients with HHD or ‘in-center HHD’ in Sweden, 70% have more frequent dialysis than thrice weekly [4]. Previous studies have shown that frequent hemodialysis may decrease the risk of cardiovascular comorbidity caused by CKD [44], which also might affect the quality of life. Moreover, the higher freedom of choice in HHD, with the possibility to choose between short daily and nocturnal regimes, might improve the possibilities to combine dialysis with work and family/social life. Although to some extent this would also apply to PD patients, freedom may be perceived even larger in HHD because you have more dialysis-free days and even higher flexibility on exactly when to perform the treatment.

Our study has strengths. It is the largest study on longitudinal changes of quality of life among dialysis patients, including a nationwide sample of patients receiving treatment with IHD, PD, and HHD. Comparing the characteristics of our study cohort to all patients in the SRR suggested that the study population was representative in the main. Moreover, we study changes in quality of life over up to 39 months using repeated measurements, and with information on several potential confounders; this is important due to the differential selection into the different dialysis modalities. Another aspect of the underlying differences between IHD, PD and HHD patients is that the reasons for drop out during the follow-up period differ thus resulting in informative censoring. In our study, like the other previous follow-up studies, the proportion of patients who died and received a kidney transplantation during the study period varied according to the dialysis modality. However, contrary to previous studies where informative censoring may have introduced bias, we handled this through joint models. We also need to acknowledge some limitations. There is a risk of residual confounding despite statistical adjustment for a variety of factors, and we cannot infer causality. Moreover, there may be misclassification bias because there were a few patients who changed dialysis modality during the follow-up period. However, as the majority of those switching modality was from HHD/PD to IHD, these changes would have biased our results mainly towards null and could likely not explain any of the observed associations. Although our sample size was large, we were still unable to analyze differences in quality of life in patients on continuous ambulatory PD as compared to those on automated PD, as well as quality of life in different IHD and HHD regimes.

In conclusion, this prospective observational cohort study of quality of life in dialysis patients receiving IHD, PD or HHD show that patients on HHD and PD patients have a higher unadjusted mean PCS compared with patients on IHD. HHD was also the only dialysis modality where patients reported increasing MCS over time. Although these results may be partly explained by residual confounding due to factors such as health literacy, they constitute an important contribution to the information to patients about what can be expected in the course of quality of life for different dialysis modalities. Such insights are instrumental for patients to make informed choices.

## Supplementary Material

sfae420_Supplemental_Files

## Data Availability

The data underlying this article will be shared on reasonable request.
