# Supplementary material for: Quality of life trajectories for different dialysis modalities—a nationwide study
Source: Clin Kidney J. 2024 Dec 20;18(2):sfae420. doi: 10.1093/ckj/sfae420 (PMC11833710; doi:10.1093/ckj/sfae420)
Supplement: sfae420_Supplemental_Files [file sfae420_supplemental_files.zip › 570 suppl figure 1_.pptx]

## Slide 1
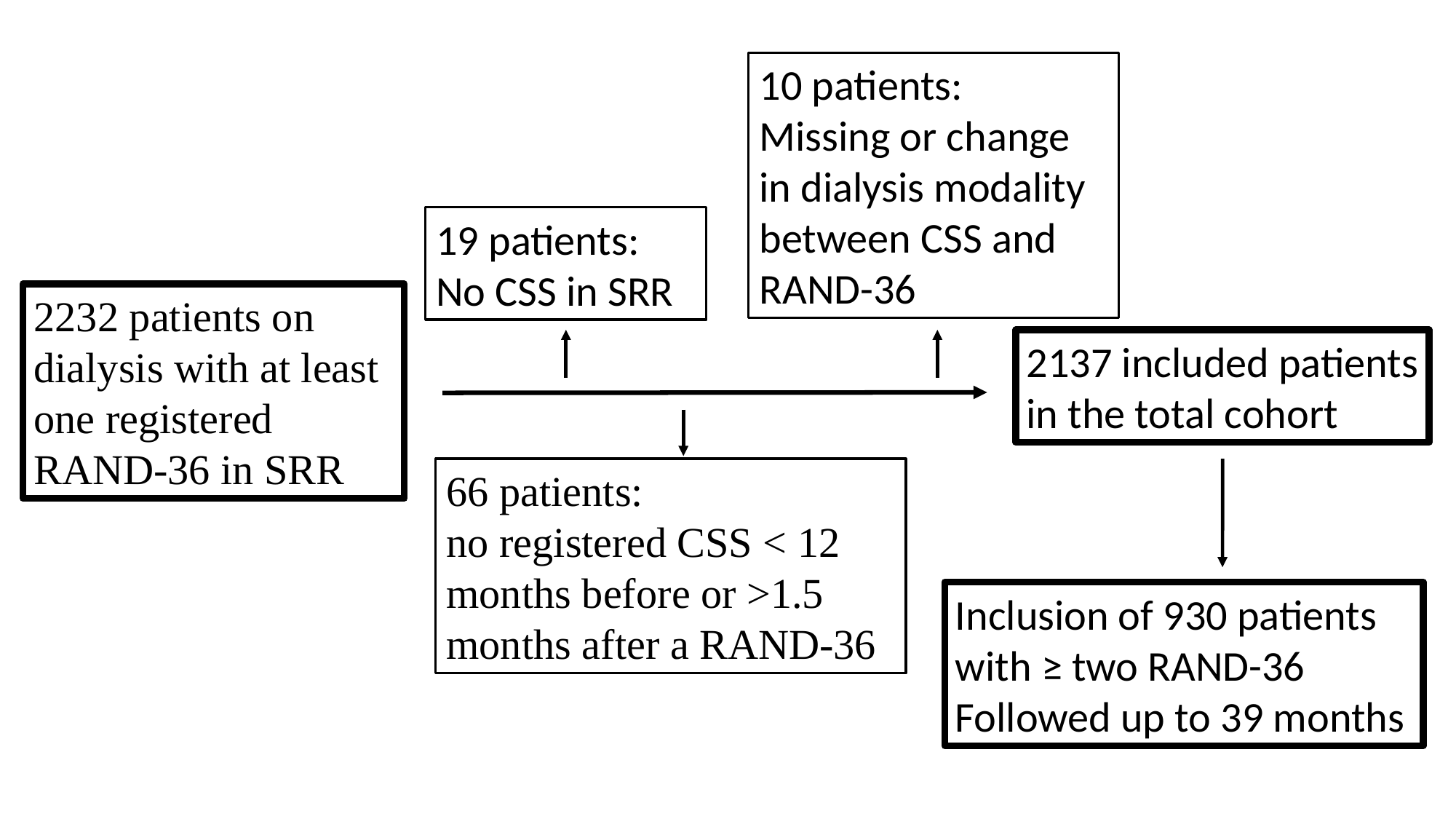

10 patients:
Missing or change in dialysis modality between CSS and RAND-36
19 patients:
No CSS in SRR
2232 patients on dialysis with at least one registered RAND-36 in SRR
2137 included patients
in the total cohort
66 patients:
no registered CSS < 12 months before or >1.5 months after a RAND-36
Inclusion of 930 patients with ≥ two RAND-36
Followed up to 39 months
