# Supplementary material for: Quality of life trajectories for different dialysis modalities—a nationwide study
Source: Clin Kidney J. 2024 Dec 20;18(2):sfae420. doi: 10.1093/ckj/sfae420 (PMC11833710; doi:10.1093/ckj/sfae420)

**SUPPLEMENT**

## **Quality of life trajectories for different dialysis modalities – a nationwide study**

Table of contents

1. Table S1. Missing data
2. Table S2. Mortality and renal transplantation among patients with IHD, HHD or PD
3. Table S3 Physical Component Score in patients on IHD and HHD (ref) or PD
4. Table S4 Mental Component Score in patients on IHD and HHD (ref) or PD
5. Table S5. Comparison of the eight different quality of life dimensions in IHD, HHD and PD
6. Table S6. Characteristics in 2137 patients with one RAND-36 measurement
7. Table S7. Comparison of Physical Component Score between patients on IHD, HHD and PD in population with one RAND-36 measurement
8. Table S8. Comparison of Mental Component Score between patients on IHD, HHD and PD in patients with one RAND-36 measurements
9. Table S9. The number of patients that changed dialysis modalities in each group
10. Table S10. Physical component score and mental component score in IHD (ref), HHD or PD among patients who did not change dialysis modality
11. Table S11; Physical component score and mental component score in IHD (ref), HHD or PD among patients with dialysis vintage less than 2 years
12. Table S12; Characteristics of prevalent patients on dialysis registered in SRR 2019-12-31
13. Figure S1. Study flow

**Table S1.** Missing data

| **Characteristics** |  | **Number (%)** | **Number (%)** | **Number (%)** |
| --- | --- | --- | --- | --- |
|  | **Total** | **HHD** | **IHD** | **PD** |
|  | 2137 | 202 | 1494 | 441 |
| **Hemoglobin, g/L** | 17 (0.8%) | 1 (0.5%) | 15 (1.0%) | 1 (0.2%) |
| **Phosphate, mmol/L** | 21 (1.0%) | 1 (0.5%) | 19 (1.3%) | 1 (0.2%) |
| **Albumin, g/L** | 28 (1.3%) | 3 (1.5%) | 24 (1.6%) | 1 (0.2%) |

**Table S3.**  Mortality and renal transplantation among patients with IHD, HHD or PD

|  | **Patients with at least one questionnaire (n=2137)** | | | | **Patients with more than one questionnaire (n=930)** | | | |
| --- | --- | --- | --- | --- | --- | --- | --- | --- |
|  | Total | IHD | PD | HHD | Total | IHD | PD | HHD |
| **Mortality,**  **n (%)** | 607 (28%) | 473  (32 %) | 103  (23 %) | 31  (16%) | 201 (22%) | 162  (23%) | 24  (19%) | 15  (17%) |
| **Renal transplantation**  **n (%)** | 281 (14%) | 129  (9 %) | 89  (20 %) | 63 (31%) | 75  (8%) | 47  (7%) | 16  (13%) | 12  (14%) |

**Table S3** Physical Component Score in patients on IHD and HHD (ref) or PD

|  | **Linear mixed model** | | **Joint model** |
| --- | --- | --- | --- |
|  | **UnadjustedUnadjusted** | **Adjusted*** | **Adjusted*** |
|  | Estimate  (95% CI) | Estimate  (95% CI) | Estimate  (95% CI) |
| **HHD (ref)** | 35.2  (33.0; 37.5) | 38.8  (30.9; 46.7) | 39.0  (31.5; 46.4) |
| **IHD#** | -4.5  (-6.9; -2.2)** | -1.8  (-4.2; 0.5) | -1.5  (-3.8; 0.7) |
| **PD#** | -2.0  (-4.9; 0.9) | -0.3  (-3.3; 2.6) | -0.2  (-3.1 ; 2.6) |
| **HHD**  **Change per year (ref)** | -0.4  (-1.5; 0.6) | -0.4  (-1.5; 0.7) | -0.3  (-1.3; 0.7) |
| **IHD##** | -0.2  (-1.3; 1.0) | -0.2  (-1.4; 0.9) | -0.4  (-1.5; 0.6) |
| **PD##** | -1.5  (-2.9; -0.04)** | -1.5  (-3.0; 0.1)** | -1.6  (-2.9; -0.2)** |

Home-hemodialysis (HHD), In-center hemodialysis (IHD), Peritoneal dialysis (PD), Confidence Interval (CI)

*Adjusted for sex, age, CCI score, diabetes, dialysis vintage, the number of days admitted to hospital the last year before baseline, and laboratory values (albumin, hemoglobin, phosphate)

**p<0.05

#Mean difference in score compared to the reference (HHD)

## Mean difference in change/year compared to reference (HHD). A positive value indicates a slower change; a negative value indicates a faster change/year.

**Table S4** Mental Component Score in patients on IHD and HHD (ref) or PD

|  | **Linear mixed model** | | **Joint model** |
| --- | --- | --- | --- |
|  | **Unadjusted** | **Adjusted*** | **Adjusted*** |
|  | Estimate  (95% CI) | Estimate  (95% CI) | Estimate  (95% CI) |
| **HHD (ref)** | 43.8 (41.2; 46.4) | 34.09 (24.9; 43.3) | 33.9 (24.9; 42.9) |
| **IHD#** | 1.8 (-1.0; 2.5) | -0.02 (-2.9; 2.8) | 0.04 (-2.7; 2.8) |
| **PD#** | 1.8 (-1.6; 5.2) | 0.2 (-3.3; 3.7) | 0.3 (-3.2; 3.8) |
| **HHD**  **Change per year (ref)** | 1.1 (-0.2; 2.5) | 1.1 (-0.3; 2.4) | 1.0 (-0.3; 2.3) |
| **IHD##** | -1.5 (-3.0; -0.1)** | -1.5 (-2.9; -0.05)** | -1.5 (-2.8; -0.2)** |
| **PD##** | -2.3 (-4.1; -0.5)** | -2.3 (-4.1; -0.5)** | -2.3 (-3.9; 0.7)** |

Home-hemodialysis (HHD), In-center hemodialysis (IHD), Peritoneal dialysis (PD), Confidence Interval (CI)

*Adjusted for sex, age, CCI score, diabetes, dialysis vintage, the number of days admitted to hospital the last year before baseline, and laboratory values (albumin, hemoglobin, phosphate)

**p<0.05

#Mean difference in score compared to the reference (HHD)

## Mean difference in change/year compared to reference (HHD). A positive value indicates a slower change; a negative value indicates a faster change/year.

**Table S5.** Comparison of the eight different quality of life dimensions in IHD, HHD and PD

|  | **Physical function** | **Physical role emotion** | **Physical pain** | **General health** |
| --- | --- | --- | --- | --- |
|  | Estimate  (95% CI) | Estimate  (95% CI) | Estimate  (95% CI) | Estimate  (95% CI) |
| **IHD (ref)** | 71.0  (51.5; 90.5) | 36.8  (11.1; 62.4) | 54.4  (33.4; 75.4) | 24.0  (9.1; 38.9) |
| **HHD#** | 6.0  (0.1; 11.8)** | 4.0  (-4.1; 12.2) | 1.0  (-5.3; 7.2) | 1.3  (-3.1; 5.8) |
| **PD#** | 4.0  (-1.2; 9.0) | 1.8  (-5.3; 8.9) | 3.7  (-1.7; 9.2) | 0.9  (-2.9; 4.8) |
| **IHD**  **Change per year (ref)** | -2.6  (-3.6; -1.7) | -0.6  (-2.2; 0.9) | -1.5  (-2.5; -0.4) | -0.6  (-1.3; 0.1) |
| **HHD##** | 2.5  (-0.3; 5.3) | 1.9  (-2.6; 6.5) | 0.3  (-2.8; 3.5) | 0.8  (-1.2; 2.9) |
| **PD##** | -3.2  (-5.6; -0.7)** | -3.6  (-7.6; 0.4) | -1.3  (-4.1; 1.4) | -1.5  (-3.4; -0.3) |

|  | **Vitality** | **Social function** | **Emotional** | **Mental** |
| --- | --- | --- | --- | --- |
|  | Estimate  (95% CI) | Estimate  (95% CI) | Estimate  (95% CI) | Estimate  (95% CI) |
| **IHD (ref)** | 36.8  (20.5; 53.0) | 43.0  (23.3; 62.7) | 63.3  (33.4; 93.2) | 50.1  (34.7; 65.6) |
| **HHD#** | 3.8  (-1.1; 8.8) | -0.4  (-6.6; 5.8) | -1.3  (-10.9; 8.2) | 3.2  (-1.5; 7.9) |
| **PD#** | 1.3  (-2.9; 5.6) | 1.5  (-3.8; 6.9) | 0.7  (-7.6; 8.9) | 1.7  (-2.4; 5.8) |
| **IHD**  **Change per year (ref)** | -1.3  (-2.1; -0.4) | -1.6  (-2.7; -0.4) | -2.2  (-3.9; -0.5) | -0.6  (-1.4; 0.1) |
| **HHD##** | 0.7  (-1.9; 3.1) | 3.3  (-0.3; 6.8) | 6.2  (0.9; 11.5)** | 1.6  (-0.6; 3.9) |
| **PD##** | -3.5  (-5.6; -1.3** | -2.9  (-6.0; 0.1) | -1.6  (-6.2; 2.9) | -1.4  (-3.4; 0.6) |

Home-hemodialysis (HHD), In-center hemodialysis (IHD), Peritoneal dialysis (PD), Confidence Interval (CI)

*Adjusted for sex, age, CCI score, diabetes, dialysis vintage, the number of days admitted to hospital the last year before baseline, and laboratory values (albumin, hemoglobin, phosphate)

**p<0.05

#Mean difference in score compared to the reference (IHD)

## Mean difference in change/year compared to reference (IHD). A positive value indicates a slower change; a negative value indicates a faster change/year.

**Table S6** Characteristics in 2137 patients with at least one RAND-36 measurement

| **Characteristic*** | **Total**  **(**2137) | **HHD**  **(202)** | **IHD**  **(1494)** | **PD (441)** |
| --- | --- | --- | --- | --- |
| **Age, years** | 70  (57 – 77) | 54  (45 – 65) | 71  (68 – 78) | 70  (57 – 77) |
| **Women** | 712 (33.3%) | 59  (29.2%) | 509 (34.1%) | 144 (32.7%) |
| **Glomerulonephritis** | 383 (1  7.9%) | 52  (25.7%) | 238 (15.9%) | 93  (21.1%) |
| **Diabetes nephropathy** | 487 (22.8%) | 28  (13.9%) | 383 (25.6%) | 76  (17.2%) |
| **Nephroangiosclerosis** | 395 (18.5%) | 53  (10.4%) | 289 (18.4%) | 104 (21.5%) |
| **Polycystic kidney disease/hereditary** | 183  (8.6%) | 29  (14.4%) | 103  (6.9%) | 51  (11.6%) |
| **Dialysis vintage, years** | 1.2  (1.3 – 1.9) | 2.0  (0.9 -4.2) | 1.4  (0.6 – 3.2) | 0.75  (0.4 – 1.6) |
| **Hemoglobin** | 113  (105 – 122) | 114  (106 - 123) | 113  (104 – 121) | 113  (160 – 124) |
| **Phosphate** | 1.6  (1.3 – 1.9) | 1.6  (1.3 – 1.9) | 1.6  (1.3 -1.9) | 1.6  (1.4 – 1.8) |
| **Albumin** | 34  (31– 37) | 37  (34 – 40) | 34.0  (32– 37) | 31  (28 – 34) |
| **C-reactive protein** | 5.0  (3.0 – 12.0) | 4  (2.4 – 8) | 5.2  (3.0 -14.0) | 5.0  (2.7 – 9.0) |
| **Charlson comorbidity index** |  |  |  |  |
| **No** | 297  (13.9%) | 55  (27.2%) | 141  (9.4%) | 101 (22.9%) |
| **Mild** | 672  (31.5%) | 67  (33.2%) | 449  (30.0%) | 156 (35.4%) |
| **Moderate** | 692  (32.4%) | 53  (26.2%) | 526  (35.2%) | 113 (25.6%) |
| **Severe** | 476  (22.3%) | 27 (13.4%) | 378 (25.3%) | 71  (16.1%) |
| **Coronary artery disease** | 641  (30.0%) | 39  (19.3%) | 491  (32.9%) | 111 (25.2%) |
| **Congestive heart failure** | 688  (32.2%) | 54  (26.7%) | 525  (35.1%) | 109 (24.7%) |
| **Peripheral vascular disease** | 278  (13.0%) | 26 (12.9%) | 216 (14.5%) | 36  (8.2%) |
| **Cerebrovascular disease** | 411  (19.2%) | 17  (8.4%) | 327 (21.9%) | 67  (15.2%) |
| **Dementia** | 11 (0.5%) | 0 (0%) | 10 (0.7%) | 1 (0.2%) |
| **Cancer** | 595  (27.8%) | 42 (20.8%) | 443 (29.7%) | 110  (24.9%) |
| **Antidepressant** | 377  (17.6%) | 25  (12.4%) | 189 (19.3%) | 63  (14.3%) |
| **Days admitted year before index** | 6 (1 – 18) | 3 (0 – 11) | 7 (1 – 22) | 4 ( 1 -12) |

*Values are presented as number (%) for categorical variables, and median, interquartile range (IQR) for continuous variables

**Table S7** Comparison of Physical Component Score between patients on IHD, HHD and PD in population with one RAND-36 measurement

|  | **Mixed model** | | **Joint model** |
| --- | --- | --- | --- |
|  | **Unadjusted** | **AdjustedAdjusted*** | **Adjusted*** |
|  | Estimate  (95% CI) | Estimate  (95% CI) | Estimate  (95% CI) |
| **IHD (ref)** | 30.2 (29.6; 30.7) | 35.5 (30.2; 40.8) | 37.4 (31.9; 42.1) |
| **HHD#** | 6.3 (4.7; 7.9)** | 2.5 (0.9; 4.1)** | 1.9 (0.4; 3.5)** |
| **PD#** | 2.5 (1.4 ; 3.7)** | 1.1 (-0.04; 2.3) | 0.9 (-0.2; 2.2) |
| **IHD**  **Change per year (ref)** | -0.5 (-0.9; -0.1) | -0.6 (-0.9; -0.2) | -0.9 (-1.3; -0.5) |
| **HHD##** | -0.2 (-1.3; 0.9) | 0.1 (-1.0;1.2) | 0.7 (-0.2; 1.8) |
| **PD##** | -1.3 (-2.2; -0.3)** | -1.2 (-2.1; -0.3)** | -0.8 (-1.7; 0.05) |

Home-hemodialysis (HHD), In-center hemodialysis (IHD), Peritoneal dialysis (PD), Confidence Interval (CI)

*Adjusted for sex, age, CCI score, diabetes, dialysis vintage, the number of days admitted to hospital the last year before baseline, and laboratory values (albumin, hemoglobin, phosphate)

**p<0.05

#Mean difference in score compared to the reference (IHD)

## Mean difference in change/year compared to reference (IHD). A positive value indicates a slower change; a negative value indicates a faster change/year.

**Table S8.** Comparison of Mental Component Score between patients on IHD, HHD and PD in patients with one RAND-36 measurements

|  | **Mixed model** | | **Joint model** |
| --- | --- | --- | --- |
|  | **Unadjusted** | **AdjustedAdjusted*** | **Adjusted*** |
|  | Estimate  (95% CI) | Estimate  (95% CI) | Estimate  (95% CI) |
| **IHD (ref)** | 44.5 (43.8; 45.1) | 29.6 (23.2; 35.9) | 29.0 (22.8; 35.1) |
| **HHD#** | -0.5 (-2.4; 1.3) | 0.8 (-1.1; 2.8) | 0.9 (-1.1; 2.7) |
| **PD#** | 0.5 (-0.8; 1.9) | 0.9 (-0.5; 2.4) | 0.95 (-0.4; 2.4) |
| **IHD**  **Change per year (ref)** | -0.1 (-0.6; 0.3) | -0.2 (-0.6; 0.2) | -0.4 (-0.8; 0.1) |
| **HHD##** | 1.2 (-0.1; 2.6) | 1.3 (-0.1; 2.6) | 1.1 (-0.2; 2.2) |
| **PD##** | -0.9 (-2.1; 0.2) | -0.9 (-2.0; 0.3) | -0.98 (-2.1; 0.02) |

Home-hemodialysis (HHD), In-center hemodialysis (IHD), Peritoneal dialysis (PD), Confidence Interval (CI)

*Adjusted for sex, age, CCI score, diabetes, dialysis vintage, the number of days admitted to hospital the last year before baseline, and laboratory values (albumin, hemoglobin, phosphate)

**p<0.05

#Mean difference in score compared to the reference (IHD)

## Mean difference in change/year compared to reference (IHD). A positive value indicates a slower change; a negative value indicates a faster change/year.

**Table S9.** The number of patients that changed dialysis modalities in each group

|  | | Change to *n* (proportion) | | |
| --- | --- | --- | --- | --- |
|  |  | HHD | IHD | PD |
| Change from | HHD | - | 24  (27 %) | 0 |
|  | IHD | 52  (7 %) | - | 8  (1 %) |
|  | PD | 3  (2 %) | 27  (21 %) | - |

**Table S10.** Physical component score and mental component score in IHD (ref), HHD or PD among patients who did not change dialysis modality

|  | **PCS** | | **MCS** | |
| --- | --- | --- | --- | --- |
|  | **Linear mixed model** | **Joint model** | **Linear mixed model** | **Joint model** |
|  | **Adjusted*** | **Adjusted*** | **Adjusted*** | **Adjusted*** |
|  | Estimate  (95% CI) | Estimate  (95% CI) | Estimate  (95% CI) | Estimate  (95% CI) |
| **IHD (ref)** | 37.5  (29.2 ; 45.9) | 38.2  (30.2 ; 46.2) | 36.7 (26.9 ; 46.5) | 36.5 (27.2 ; 45.8) |
| **HHD#** | 4.0  (1.2 ; 6.8)** | 3.6  (0.9 ; 6.2)** | 0.6 (-2.6 ;3.9) | -0.03 (-2.9 ; 2.7) |
| **PD#** | 2.4  (0.04 ; 4.7) | 2.2  (-0.02 ; 4.4) | 0.7 (-2.6 ; 3.9) | 0.3 (-2.9 ; 2.9) |
| **IHD**  **Change per year (ref)** | -0.8  (-1.2; -0.4) | -0.9  (-1.2; -0.5) | -0.4 (-0.9 ; 0.04) | -0.5 (-0.9;-0.08) |
| **HHD##** | 0.4  (-0.9 ; 1.8) | 0.7  (-0.3 ; 1.9) | 2.1 (0.4 ; 3.8)** | 2.2 (0.7 ; 3.6)** |
| **PD##** | -0.9  (-2.1 ; 0.24) | -0.8  (-1.9 ; 0.3) | -1.3 (-2.8 ; 0.1) | -1.3 (-2.6 ; 0.3) |

**p-value<0.05 compared to IHD

**Table S11;** Physical component score and mental component score in IHD (ref), HHD or PD among patients with dialysis vintage less than 2 years

|  | **PCS** | | **MCS** | |
| --- | --- | --- | --- | --- |
|  | **Linear mixed model** | **Joint model** | **Linear mixed model** | **Joint model** |
|  | **Adjusted*** | **Adjusted*** | **Adjusted*** | **Adjusted*** |
|  | Estimate  (95% CI) | Estimate  (95% CI) | Estimate  (95% CI) | Estimate  (95% CI) |
| **IHD (ref)** | 37.6  (27.7; 47.5) | 37.9  (28.2; 47.7) | 25.3  (14.2; 36.4) | 25.1 (14.7; 35.6) |
| **HHD#** | 1.6  (-1.6; 4.8) | 1.3  (-1.9; 4.4) | -0.4 (-4.2; 3.4) | -0.3 (-4.0; 3.2) |
| **PD#** | 2.1  (-0.28; 4.5) | 1.9  (-0.4; 4.2) | 0.5 (-2.3; 3.3) | 0.6 (-2.1; 3.2) |
| **IHD**  **Change per year (ref)** | -0.2  (-0.7; 0.3) | -0.2  (-0.7; 0.2) | -0.5 (-1.1; 0.2) | -0.5 (-0.04; 0.1) |
| **HHD##** | 0.3  (-1.3; 1.9) | 0.5  (-0.9; 1.9) | 2.1 (0.4; 3.8) | 1.3 (-0.5; 3.1) |
| **PD##** | -1.6  (-2.8; -0.5)** | -1.4  (-2.4; 0.4) | -1.3 (-2.8; 0.1) | -0.6 (-1.8; 0.7) |

**p-value<0.05 compared to IHD

**Table S12;** Characteristics of prevalent patients on dialysis registered in the cross-sectional survey in the SRR 2019-09-15 until 2019-10-15.

|  | **IHD cohort** | **IHD SRR** | **HHD cohort** | **HHD SRR** | **PD cohort** | **PD SRR** |
| --- | --- | --- | --- | --- | --- | --- |
| Age (median) | 72 | 68 | 55 | 56 | 71 | 67 |
| Women (%) | 33 | 36 | 36 | 32 | 30 | 29 |
| Diabetic nephropathy (%) | 24 | 27 | 18 | 16 | 19 | 22 |
| Glomerulonephritis (%) | 17 | 14 | 25 | 28 | 19 | 20 |
| Nephroangiosclerosis (%) | 21 | 19 | 11 | 8 | 24 | 23 |
| Polycystic kidney disease (%) | 7 | 7 | 9 | 10 | 11 | 6 |

Supplementary figure 1

Study flow


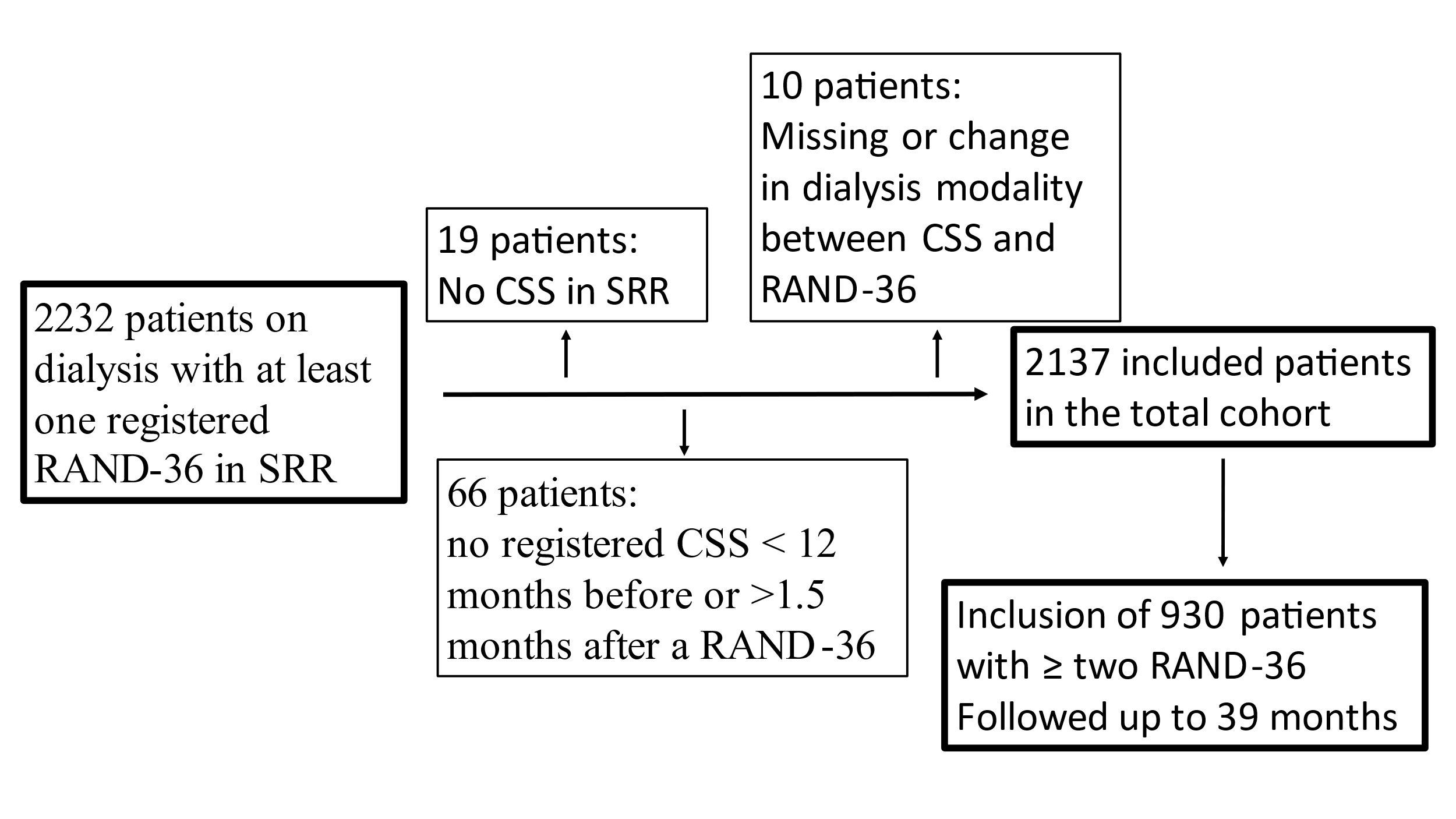

Supplement: sfae420_Supplemental_Files [file sfae420_supplemental_files.zip › 570 Supplement QoL 240901.docx]
